# Preliminary Data on the Interaction between Some Biometals and Oxidative Stress Status in Mild Cognitive Impairment and Alzheimer's Disease Patients

**DOI:** 10.1155/2017/7156928

**Published:** 2017-07-24

**Authors:** Ioana-Miruna Balmuș, Stefan-Adrian Strungaru, Alin Ciobica, Mircea-Nicusor Nicoara, Romeo Dobrin, Gabriel Plavan, Cristinel Ștefănescu

**Affiliations:** ^1^Department of Biology, Faculty of Biology, “Alexandru Ioan Cuza” University of Iasi, Carol I Avenue, 20A, 700505 Iasi, Romania; ^2^Department of Research, Faculty of Biology, “Alexandru Ioan Cuza” University of Iasi, Carol I Avenue, 20A, 700505 Iasi, Romania; ^3^Department of Psychiatry, Faculty of Medicine, “Gr. T. Popa” University of Medicine and Pharmacy, 16th University Avenue, 700115 Iasi, Romania

## Abstract

Increased interest regarding the biometal mechanisms of action and the pathways in which they have regulatory roles was lately observed. Particularly, it was shown that biometal homeostasis dysregulation may lead to neurodegeneration including Alzheimer's disease, Parkinson disease, or prion protein disease, since important molecular signaling mechanisms in brain functions implicate both oxidative stress and redox active biometals. Oxidative stress could be a result of a breakdown in metal-ion homeostasis which leads to abnormal metal protein chelation. In our previous work, we reported a strong correlation between Alzheimer's disease and oxidative stress. Consequently, the aim of the present work was to evaluate some of the biometals' levels (magnesium, manganese, and iron), the specific activity of some antioxidant enzymes (superoxide dismutase and glutathione peroxidase), and a common lipid peroxidation marker (malondialdehyde concentration), in mild cognitive impairment (*n* = 15) and Alzheimer's disease (*n* = 15) patients, compared to age-matched healthy subjects (*n* = 15). We found increased lipid peroxidation effects, low antioxidant defense, low magnesium and iron concentrations, and high manganese levels in mild cognitive impairment and Alzheimer's disease patients, in a gradual manner. These data could be relevant for future association studies regarding the prediction of Alzheimer's disease development risk or circling through stages by analyzing both active redox metals, oxidative stress markers, and the correlations in between.

## 1. Introduction

It is now generally accepted that several biometals (BM) such as iron, copper, zinc, manganese, and magnesium are vital in the complex cellular activities and regulation [1–5]. Lately, there is an increased interest regarding the BM mechanisms of action due to their potential to lead to several pathway degeneration when homeostatically impaired. In this way, neurodegenerative diseases such as Alzheimer's disease (AD), Parkinson disease, and prion protein disease are shown to be closely related to several BM levels.

AD is a complex disorder involving both behavioral and molecular distress. In this way, it is now accepted that AD is a multifactorial disease in which several important components have been described: the discrete biochemical changes which firstly occur triggering cellular modifications such as amyloid accumulation and neurofibrillary tangles formation; the histological typical features accompanied by synaptic disruption and neuronal loss; and last but not the least the visible symptoms of the behavioral component—memory loss, cognitive decline, and related comorbidities (affective distress and somatic disorders such as chronic pain or anemia) [6–9]. Also, considering these findings, it is now accepted that mild cognitive impairment (MCI) is a disorder providing a major risk factor for AD [10]. Moreover, while some overlapping traits between MCI and the early stages of AD in considering the characteristic mild cognitive decline were suggested, it was shown that AD cognitive abilities gradually decline, but MCI patients' cognitive state remains stable for years [10].

Oxidative stress is also a major component of AD pathology. The common knowledge on aging now includes a biochemical theory that partly explains the cellular decline due to oxidative/antioxidant process imbalance occurrence at a cellular level as we age. Together with that, the thorough description of brain biochemical mechanisms which revealed its high oxygen resources needs and its special membrane lipid-rich structure leads to the conclusion that brain tissue is extremely susceptible to oxidative stress. In this context, it was demonstrated that oxidative stress plays important roles in AD pathology, both at its first molecular changes and also during its development up to its final stages.

Also, oxidative stress may also be a result of a breakdown in metal-ion homeostasis which leads to abnormal metal protein chelation. Extensive evidence points to an important implication of several both toxic and redox metal ions which can contribute to DNA and protein damage causing oxidative stress and molecular damage (as reviewed by [11]) by being involved in cycles of electron transfer reactions from and to the substrates which make them extremely important in redox and metal homeostasis, both of which are tightly related [12]. Therefore, any ionic metal unbalance occurring at the cellular or peripheral level is reflected in abnormal redox homeostasis followed by excessive reactive oxygen species (ROS) production, oxidative stress, and their further effects [13].

Furthermore, the critical role of copper, iron, and other trace redox-active transition metals was shown recently to be implicated in the pathogenesis of AD [14, 15]. While our group previously suggested a strong link between oxidative stress and Alzheimer's disease [16, 17], we aimed to assess the possible cause/effect relationship between BM abnormal levels dynamics and the increased oxidative damage occurring in AD pathology. Moreover, we demonstrated a progressive pattern of oxidative markers change during both mild cognitive impairment and Alzheimer's disease patients analysis which could also be linked to a progressive BM level pathological tendency.

Consequently, the aim of the present work was to evaluate some relevant BM levels (magnesium, manganese, and iron), the specific activity of some antioxidant enzymes (superoxide dismutase SOD and glutathione peroxidase (GPx)), and malondialdehyde (MDA) levels as a marker of lipid peroxidation, in MCI and AD patients, compared with age-matched healthy subjects.

## 2. Materials and Methods

### 2.1. Patient Recruitment

In this case-control study, we collected blood samples from 30 patients with AD (15 patients) and MCI (15 patients). The control group was consisted from healthy age- and sex-matched participants (*n* = 15). All the participants were recruited from the “Socola” Regional Institute of Psychiatry (Iasi, Romania) based on ethical agreement from the Regional Institute of Psychiatry Board Committee. Also, the cognitive status of the participants was assessed using standard Mini-Mental State Examination (MMSE) [18] and Alzheimer's Disease Assessment Scale-Cognitive (ADAS-Cog) [19]. All the AD patients underwent psychiatric diagnosis fulfilling NINCDS ADRDA criteria [20, 21]. Also, MCI diagnosis followed Petersen et al. criteria (memory impairment accompanied by general cognitive and functional abilities preservation) [22]. The study was conducted according to Helsinki Declaration and national and European regulations on Biomedical Research. All the patients or their families were given and signed a written informed consent for their contribution in this study. Several exclusion criteria were provided for patients' recruitment such as antioxidant supplementation and acute or severe comorbidities.

### 2.2. Sample Preparation

Blood samples have been collected before breakfast and allowed to clot. After centrifugation (3000 rpm, 15 minutes, 4°C), blood sera were separated, aliquoted, and stored at −22°C until analysis. For BM quantification, the stored aliquots were processed according to the following protocol: 1 ml of sample was digested with 3 ml nitric acid 65% and 2 ml of hydrogen peroxide in decontaminated TFM pressure vessels that were inserted in Speedwave MWS-2 produced by Berghof. The digestion program for samples was in steps as follows: 145°C for 5 min, 190°C for 10 min, and 100°C for 10 min [23]. After the microwave digestion, the samples were transferred in 25 ml decontaminated flasks and filled up to volume with ultrapure water. No special preparation except for the biochemical analysis kit brochures' mentions were needed for the biochemical analysis protocols.

### 2.3. Biometals Separation and Quantification

High-purity and producer-certified-quality reagents were used for the element separation and measurement. Ultrapure water filtered by LaboStar™3/7 TWF (Siemens) purification system from double-distilled water was used for decontamination, sample preparation, and reagent dilution. High-purity nitric acid 65% (Merck, Germany) and hydrogen peroxide EMSURE 30% stabilized for higher storage temperature (Merck, Germany) were used in the metal digestion process from the biological samples. The standard stock solutions for AAS used in the calibration method were certified by Merck, Germany. All the necessary solution used in calibration and quantification of the metals were manganese (1000 mg/l), iron (1000 mg/l), and magnesium (1000 mg/l).

Atomic absorption spectrometer with a high-resolution continuum source equipped with graphite furnace and platform, ContrAA 600 from Analitik Jena, Germany, was used for all the element measurements. Matrix sample modifiers diluted from certified solutions of Pd/Mg(NO₃)₂ (Merck Germany) were necessary. Blind samples for testing any possible contamination from laboratory and reagents were prepared. Standard solutions were used for quality-control sample preparation consisting in various successive concentrations.

### 2.4. Biochemical Analysis

Biochemical analysis included SOD, GPx, and MDA quantification. SOD enzymatic activity was determined as enzymatic reaction inhibition rate using a spectrophotometric SOD Assay Kit (Sigma, Germany) according to the manufacturer's instructions. The method is based on the WST (water-soluble tetrazolium) salt reaction with superoxide anion producing a water-soluble formazan dye. Therefore, indirect measurement of SOD activity is obtained by analyzing the linear correlation with the rates of xanthine oxidase catalyzed O_2_ reduction which is inhibited by SOD. Following this measurement, the results were normalized by total sera protein concentration and transformed in SOD activity units. GPx activity was determined using GPx Cellular Activity Assay Kit CGP-1 (Sigma, Germany). This kit also uses an indirect determination method based on the oxidation of glutathione to oxidized glutathione coupled with the inverse reaction in the presence of GPx, glutathione reductase, and NADPH as an enzymatic cofactor. The method is therefore based on the NAPDH concentration decrease measurement in the reaction media, correspondent to the GPx activity during which NADPH is oxidized to NADP^+^. MDA levels were assessed using thiobarbituric acid-reactive substances (TBARs) determination method. Trichoracetic acid (50%, 0.5 ml) and thiobarbituric acid (0.73%, 0.55 ml) were mixed with blood sera (0.1 ml) and vortexed. Afterwards, a 20-minute incubation at 100°C (boiling water bath) and a 10-minute centrifugation (3000 rpm) were performed. The supernatants were read at 532 nm and the absorbances were read against MDA standard curve (the results were expressed as nmol MDA/ml blood serum) [24, 25].

### 2.5. Statistical Analysis

Firstly, the Shapiro-Wilk normality test for the data sets was performed to study the samples' distribution. One-way ANOVA followed by the Tukey HSD test was performed to demonstrate the significant variance of each metal concentration in blood serum between studied groups. All the statistical analyses were carried out by using OriginPro v.9.3 (2016) software created by OriginLab Corporation, USA. The results were reported as means ± standard error of the means. *F* values for which *p* < 0.05 were regarded as statistically significant. Pearson's correlation coefficient and regression analysis were used to evaluate the connection between antioxidant defense, lipid peroxidation, and BM serum levels for which Minitab 17 (Minitab Inc., 2013) application was used.

## 3. Results

In the present study, we measured several important BM levels, in the peripheral blood of MCI and AD patients, compared with age- and sex-matched controls. Chemical analysis of the subjects' blood serum revealed significant differences regarding the magnesium, manganese, and iron levels in the MCI and AD patients' sera.

Data on patients and oxidative stress markers have been previously described [26] but are summarized in Table 1.

**Figure 1:**
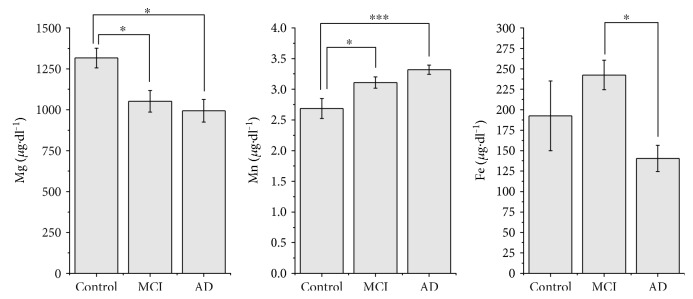
Magnesium, manganese, and iron concentrations in blood sera (presented as mean ± SEM) in studied groups (^∗^*p* < 0.05 and ^∗∗∗^*p* < 0.001, Tukey HSD test).

We observed an iron levels decrease in the AD patients sera (140.43 ± 16.02 *μ*g/dl) as compared with the healthy sex- and age-matched controls (192.45 ± 42.51 *μ*g/dl). Also, interestingly, we firstly observed a slight increase of the iron levels in the MCI patients (242.47 ± 18.06 *μ*g/dl) followed by the significant decrease in AD patients (Figure 1). Overall one-way ANOVA showed a significant variation between the groups: C – MCI − AD [F(2, 30) = 3.82; *p* = 0.033]. Post hoc comparisons using the Tukey test indicated that the mean score for the AD group (*M* = 140.43, SD = 57.77) was significantly different than the MCI group (*M* = 242.47; SD = 57.12).

Regarding the manganese levels, we observed a statistically significant increase of manganese levels in AD patients' sera (3.32 ± 0.07 *μ*g/dl, *p* < 0.001) and MCI patients' sera (3.10 ± 0.09 *μ*g/dl, *p* < 0.05) as compared to the healthy sex- and age-matched controls' sera (2.68 ± 0.16 *μ*g/dl) (overall ANOVA: F(2, 32) = 8.93, *p* = 0.0008) (Figure 1). Post hoc analysis revealed that the mean score for the MCI and AD groups were significantly different from the control group regarding the manganese levels and similarly regarding the magnesium levels.

Contrarily, we observed that AD patients' (994.08 ± 69.04 *μ*g/dl, *p* < 0.01) and MCI patients' (1051.40 ± 65.43 *μ*g/dl, *p* < 0.05) magnesium levels tend to decrease as compared to those of healthy controls (1316.46 ± 60.27 *μ*g/dl) (Figure 1). Also, overall one-way ANOVA analysis revealed a significant pattern of magnesium level decrease [F(2, 35) = 4.73; *p* = 0.015] for the experiment groups, as compared with the healthy sex- and age- matched controls.

**Figure 2:**
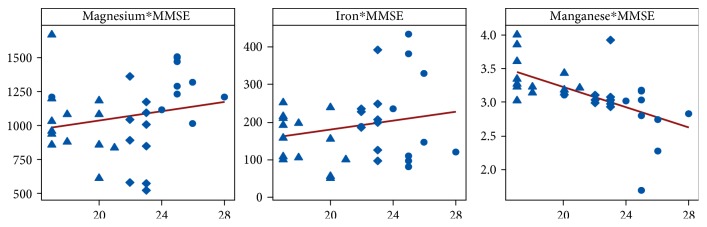
Correlation between MMSE scores and BM levels in control (●), MCI (♦), and AD (▲) patients (explanation in text).

Regarding the BM levels trending, several direct moderate and low correlations (post hoc Pearson's correlation) have been observed. In this way, while analysing iron against manganese trending, a moderate negative correlation has been observed (Fe-Mn: *r* = −0.431, *p* = 0.012). Weak negative correlation was obtained while analysing magnesium versus manganese trending (Mg-Mn: *r* = −0.27, *p* = 0.12), and no statistical correlation was found for iron versus magnesium comparison. However, while comparing BM levels with specific psychiatric scales applied to the patients, we observed a strong negative correlation between MMSE score and manganese levels (*r* = −0.585, *p* < 0.001) (Figure 2).

Interesting results were obtained in post hoc analysis (Pearson correlation) of biometal concentrations versus oxidative stress status correlations. In this way, we obtained significant statistical correlations for two of the oxidative stress markers as compared with manganese concentrations (Mn-GPx: *r* = −0.564, *p* < 0.001; Mn-MDA: *r* = 0.561, *p* < 0.001). For magnesium levels, moderate statistical correlations were obtained for Mg-GPx (*r* = 0.509*p* = 0.002) and Mg-MDA (*r* = −0.383, *p* < 0.05) comparisons. No statistical significant correlations were found during post hoc analysis of serum iron levels and oxidative stress markers.

## 4. Discussion

The present work aimed to evaluate manganese, magnesium, and iron levels, the specific activity of some antioxidant enzymes (SOD and GPx), and MDA levels as a marker of lipid peroxidation therefore cellular damage, in MCI and AD patients, compared to age-matched healthy subjects. We found increased lipid peroxidation effects, low antioxidant defense, low magnesium and iron concentrations, and high manganese levels in MCI and AD patients.

Previous research suggested some contradictory results concerning the BM serum levels in neurodegenerative disorders. Recent studies reported various tendencies in BM level dynamics in demented patients [16, 26, 27]. In this way, Barbagallo et al. [28] showed decreased magnesium ion levels in AD patients' serum. Also, Cilliler et al. [29] reported a correlation between low AD group magnesium levels and MMSE score. Similarly, manganese level analysis in the recent studies revealed controverted results. The most recent meta-analysis on the matter, Du et al. [30], reports controversial dynamics of manganese levels presenting several studies which show a significant decrease of this parameter in demented patients' sera, while in other studies, no difference or significant increase were obtained. We also found statistical correlations between MMSE scores versus manganese levels and ADAS-COG versus magnesium and manganese levels (data not shown). Regarding the iron levels, the previous studies suggest no significant change in demented patients [31], while the more recent Paglia et al. [32] study reported a progressive decrease of this parameter in AD, MCI patients, subjective memory complaint, and healthy participants. In our study, we report similar tendencies of the discussed BM in MCI and AD patients, as compared to age- and sex-matched control group and also significant statistical correlations for all the studied biometals versus lipid peroxidation marker, moderate statistical correlations for antioxidant enzymes versus magnesium levels, and no statistical significant correlation between oxidative stress markers and iron levels. However, an interesting iron level pattern of progression was observed. Previous studies show high specific antioxidant enzymes activities [33–35] and a compensatory activity in the body. Also, several studies reported decreased nonenzymatic antioxidant factor levels and high iron, aluminum, and mercury concentrations in demented patients [36]. Furthermore, clinical trials showed increased oxidative stress markers' levels in patients with MCI as well [37–39].

Similar iron pattern of variation was reported by Paglia et al. [32] who showed that the iron level increase (in individuals showing subjective memory complaint) is followed by abrupt concentration decrease in MCI and AD patients. These findings, together with the study of Smith et al. [40] which show increased iron levels in AD and MCI brains forming iron deposits could suggest that anemia may be a common condition found in AD patients. This idea was also suggested by Hare et al. [41] who reported low iron plasma levels in correlation with low-bound transferrin levels. Moreover, our findings could be explained by the importance of iron in oxidative stress modulation. While high levels of iron were observed in demented patients' brain and high oxidative stress is a common trait for AD, the low-iron serum levels could suggest that intense brain redox activity is resulting in excess iron concentrations in that tissue. This may be the reason why an increased iron-modulated immune response could be observed in the brain tissue during initial accumulation of beta-amyloid plaques [42]. However, while our previous results show increased serum oxidative stress levels, it seems that this variation is not dependent on iron activity.

Although controversial results were obtained in similar studies which showed that manganese levels in demented patients are decreased, many reports demonstrate that manganese could contribute to beta-amyloid plaque formation [43] and therefore an increase of this parameter would be possible both in brain tissue and also in blood serum. In this way, Tong et al. reported a plasma A*β* peptides concentration increase in correlation with high serum manganese levels. Although manganese was shown to significantly increase hippocampal glutathione peroxidase (GPx) activity [44] and manganese-dependent SOD (SOD2) [45, 46], it seems that an inverse correlation may be observed in blood sera of demented patients regarding GPx activity and total SOD activity. However, Dobson and Aschner [47] extensively discuss the oxidative stress induction potential of excess manganese. Many redox activity molecular studies on effects of manganese accumulation led to mitochondrial oxidative stress pathway description. Similar to other toxicants, it seems that excessive manganese could lead mitochondrial electron transport chain perturbance, which eventually causes additional ROS, cellular oxidative stress, and furtherly apoptosis. Also, in the redox reactivity series of metals, manganese occupies a leading position; therefore, it possesses the highest potential to generate ROS. In this way, excessive manganese intake may lead to mitochondrial dysfunction and oxidative stress alongside neuronal and astrocytic apoptosis. This aspect could be observed in the strong positive correlation between manganese and MDA levels which reflects the effects of excessive ROS production through molecular level causing lipid peroxidation and cellular damage.

The role of magnesium deficiency in AD pathogenesis has been extensively discussed [48]. Moreover, Durlach [49] brings several more arguments on magnesium mechanisms of modulation in glutamatergic transmission and hippocampal activity. Also, in an AD mouse model, Li et al. [50] points to imminent recovery of cognitive deficits and synaptic loss following magnesium administration. Xu et al. [51] reported that magnesium deficiency in rats may lead to increased free radical oxygen species, also introducing the magnesium implication in inflammation and oxidative lesions. In this way, the correlation between magnesium and the two antioxidant enzymes (GPx and SOD) is entitled since the high rate of ROS production in the presence of low antioxidant activity could be a result of magnesium depletion.

## 5. Conclusions

Our data show progressive increase of manganese levels in demented patients, as compared with healthy controls and progressive decrease of magnesium levels in the groups. A slight increase in iron serum levels was observed in MCI patients followed by an abrupt level drop in AD patients. We observed several strong correlations between cognitive status and biometals serum levels. Also, several correlations between the studied biometal sera levels and main oxidative stress markers were observed. Positive correlations were found in manganese versus MDA, magnesium versus GPx, and magnesium versus SOD analysis and negative correlations in manganese versus SOD and manganese versus GPx analysis. Still, no correlations were found between serum iron levels and serum oxidative stress markers. These data could be relevant for future association studies regarding the prediction of AD development risk or cycling through stages by analyzing both active redox metals and oxidative stress markers, and the correlations in between.

## Figures and Tables

**Table 1:** Demographics, functional description, and oxidative marker assessment of the study groups.

	Control, *n* = 15	MCI, *n* = 15	AD, *n* = 15
Age (means ± SEM, years)	62.5 ± 3.4	63.2 ± 4.2	65.8 ± 3.9
Sex (% F/% M)	46.6% F/53.3% M	33.3% F/66.6% M	40% F/60% M
MMSE score (means ± SEM)	26 ± 0.5	22.2 ± 0.3	18.5 ± 0.3
SOD (U/ml)	0.22 ± 0.008	0.18 ± 0.001^∗∗∗^	0.17 ± 0.003^∗∗∗^
GPx (U/ml)	0.142 ± 0.001	0.049 ± 0.002^∗∗∗^	0.045 ± 0.001^∗∗∗^
MDA (nmol/l)	9008 ± 0.14	13.141 ± 0.36^∗∗∗^	18.158 ± 0.26^∗∗∗^

Results are presented as mean ± SEM; ^∗∗∗^*p* < 0.001, as compared to the control group.
